# Sweet dreams could be made of this: carbohydrate‐responsive element‐binding protein (ChREBP) as a target for hepatocellular carcinoma therapy

**DOI:** 10.1002/1878-0261.13669

**Published:** 2024-06-04

**Authors:** Maite G. Fernández‐Barrena, Matías A. Avila

**Affiliations:** ^1^ Hepatology Laboratory, Solid Tumors Program, CIMA, CCUN University of Navarra Pamplona Spain; ^2^ CIBERehd Instituto de Salud Carlos III Madrid Spain; ^3^ Instituto de Investigaciones Sanitarias de Navarra IdiSNA Pamplona Spain

**Keywords:** ChREBP, hepatocellular carcinoma, metabolic reprogramming, PI3K‐AKT signaling, therapy

## Abstract

Rewiring of cellular metabolism is now fully recognized as a hallmark of cancer. Tumor cells reprogram metabolic pathways to meet the energetic and macromolecular demands to support unrestricted growth and survival under unfavorable conditions. It is becoming apparent that these adaptations underpin most of the traits that define a cancer cell's identity, including the ability to avoid immune surveillance, endure nutrient and oxygen restrictions, detach and migrate from their natural histological niche, and avert human‐made aggressions (*i.e.*, therapy). In a recent study, Benichou and collaborators identify carbohydrate‐responsive element‐binding protein (ChREBP), a master regulator of physiological glucose metabolism, as an oncogene in hepatocellular carcinoma (HCC) development. Upregulation of ChREBP expression results in a self‐stimulatory loop interconnecting PI3K/AKT signaling and glucose metabolism to feed fatty acid and nucleotide synthesis supporting tumorigenesis. Importantly, pharmacological inhibition of ChREBP activity quells *in vivo* HCC tumor growth without causing systemic toxicity. This study identifies novel oncometabolic pathways and open up new avenues to improve the treatment of a deadly tumor.

## Graphical abstract

ChREBP serves as a molecular link between growth signals (PI3K/AKT) and the activation of intertwined metabolic pathways essential for neoplastic cell growth and proliferation. ChREBP overexpression in HCC rewires glycolysis, the pentose phosphate pathway, and nucleotide biosynthesis through p85α upregulation. Pharmacological targeting of ChREBP inhibits experimental hepatocarcinogenesis.

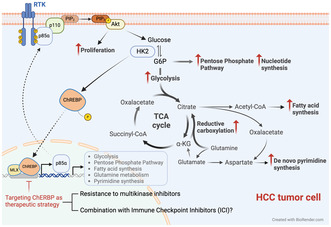

**Abbreviations**
ChIP: chromatin immunoprecipitation
ChREBP: carbohydrate‐responsive element‐binding protein
G6P: glucose 6‐phosphate
HBV: hepatitis B virus
HCC: hepatocellular carcinoma
HCV: hepatitis C virus
HK2: hexokinase 2
ICI: immune checkpoint inhibitors
MASLD: metabolic dysfunction associated steatotic liver disease
MLX: max‐like protein
PI3K: phosphoinositide 3‐kinase
PPP: pentose phosphate pathway
VEGF: vascular endothelial growth factor

Hepatocellular carcinoma (HCC) is the most frequent primary liver malignancy. HCC mainly develops on a background of chronic liver injury and inflammation, conditions associated with the major etiological factors of this neoplasia, which are persistent viral infection (hepatitis B and C viruses, HBV and HCV), alcohol abuse, and metabolic dysfunction‐associated steatotic liver disease (MASLD) [1]. In spite of significant recent advances in the management of HCC, the 5‐year survival rate of these patients is still below 20%, and yearly incidence and mortality rates are almost equal [2]. This dismal situation is due to several factors, including a frequent late diagnosis when patients are not amenable to curative surgical or loco‐regional therapies, and to the lack of response to conventional systemic chemotherapies [2]. The advent of targeted therapies such as the multikinase inhibitor sorafenib resulted in a measurable albeit modest advance in patients' survival [2]. However, more recently, the combination of immune checkpoint inhibitors (ICI) with anti‐vascular endothelial growth factor (VEGF) antibodies was proven clinically superior to sorafenib [2]. Still, the majority of patients with advanced HCC experience therapeutic resistance and disease progression. To overcome this situation, a better understanding of the pathogenic mechanisms of HCC is essential.

Comprehensive molecular profiling studies performed over the past decade have identified molecular HCC subclasses characterized by particular genetic alterations, the activation of specific signaling pathways, and differential proliferative activity and aggressiveness [3]. In parallel to these molecular alterations, one overarching feature of cancer cells is their metabolic reprogramming to meet the energetic and macromolecular demands to sustain growth and survival in unfavorable conditions, including therapeutic pressure [4, 5]. While metabolic reprogramming is observed across all tumor cell types, the extensive role played by hepatocytes in intermediary metabolism makes this adaptation particularly relevant in HCC [6]. The identification of metabolic traits critical for the growth of cancer cells may lead to new therapeutic strategies ideally devoid of toxicity for normal cells. In a recent study, Benichou and colleagues uncovered the transcription factor carbohydrate‐responsive element‐binding protein (ChREBP) as a robust candidate for pharmacological targeting in HCC [7].

ChREBP is a glucose responsive transcription factor extensively characterized as a master physiological regulator of multiple liver metabolic pathways, including glycolysis, the pentose phosphate pathway (PPP), and lipogenesis [8]. Increased intracellular glucose levels result in glucose 6‐phosphate (G6P) accumulation, and G6P triggers ChREBP activation and nuclear translocation, where together with Max‐like protein (MLX), it drives the expression of a broad complement of glucose‐responsive genes [8]. Previous studies reported the overexpression of ChREBP and MLX in human HCC, and demonstrated that the independent deletion of either gene in hepatocytes hampered the development of HCC in different experimental models [9, 10, 11]. Now, Benichou and coworkers confirm the transcriptional upregulation of ChREBP in several HCC datasets, and demonstrate the correlation between high ChREBP mRNA levels and a poor patients' prognosis. Notably, ChREBP overexpression was related to tumor etiology, being observed in those associated with MASLD and HCV infection, but not in cancers related to alcohol abuse or HBV infection. One key finding in Benichou's report was that the stable hepatic overexpression of ChREBP in mice was sufficient to induce HCC development with 100% penetrance, strongly supporting an oncogenic role for ChREBP in the liver. Transcriptional analyses of ChREBP‐driven tumors identified genes involved in metabolic processes, cell proliferation, and dedifferentiation, characteristic of human HCCs with more aggressive phenotypes. Mechanistic studies, including chromatin immunoprecipitation (ChIP)‐sequencing assays, revealed that in the context of high glucose concentrations, ChREBP overexpression stimulated the transcription of the *Pik3r1* gene. *Pik3r1* codes for p85α, the regulatory subunit of phosphoinositide 3‐kinase (PI3K) in the PI3K‐AKT signaling pathway, known to be upregulated in HCC and contribute to metabolic reprogramming and cancer progression [12]. ChREBP‐mediated p85α upregulation enhanced PI3K‐AKT signaling in response to growth factors and, importantly, liver tumorigenesis induced by ChREBP overexpression was shown to depend on p85α expression. One remarkable finding of Benichou and colleagues was the identification of a positive feedback loop by which ChREBP‐triggered p85α‐PI3K‐AKT signaling leads to enhanced ChREBP nuclear translocation and activation. This self‐amplifying circuit relied on the accumulation of the ChREBP activator G6P due to the upregulation of the oncogenic enzyme hexokinase 2 (HK2), responsible for G6P synthesis in tumor cells [4]. These findings reinforce the notion of ChREBP as a key player in HCC metabolic reprogramming.

Moreover, through a series of elegant *in vitro* and *in vivo* experiments involving isotopic labeling and flux analyses, the authors clearly demonstrate how ChREBP rewires glucose metabolism toward glycolysis and *de novo* lipogenesis, and also targets glucose and glutamine to feed the PPP pathway and pyrimidine synthesis in the context of cancer cells (Fig. 1). Notably, the authors demonstrate for the first time how ChREBP activation is responsible for increased glutamine reductive carboxylation, another important cellular carbon source for *de novo* lipogenesis, especially during impaired mitochondrial function as occurs in HCC [13]. Most interestingly, by implementing clinically relevant genetic and pharmacological approaches, evidence is provided supporting the therapeutic potential of targeting ChREBP activity to quell HCC progression and reverse sorafenib resistance.

**Fig. 1:**
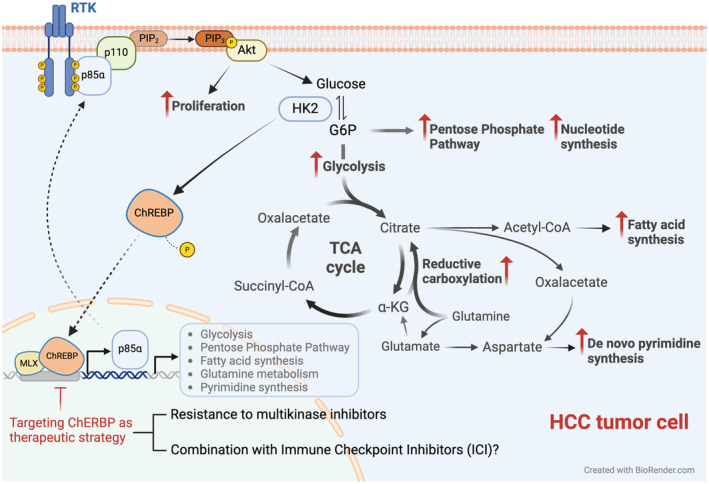
Mechanisms involved in ChREBP‐mediated hepatocarcinogenesis. The glucose responsive transcription factor ChREBP is overexpressed in hepatocellular carcinoma (HCC) cells. ChREBP connects growth factor signaling from tyrosine kinase receptors (RTK) and the phosphatidylinositol 3‐kinase‐AKT (PI3K/AKT) cascade with the activation of metabolic pathways essential for neoplastic cell growth and proliferation. ChREBP overexpression in HCC rewires glycolysis, the pentose phosphate pathway and nucleotide biosynthesis through p85α upregulation. Pharmacological targeting of ChREBP inhibits experimental hepatocarcinogenesis. HK2, hexokinase 2; TCA, tricarboxylic acid cycle.

All in all, the remarkable study of Benichou and collaborators conveys a robust fundamental message with clear translational implications. However, identifying ChREBP as such a key player in the pathogenesis of HCC certainly poses additional questions.

One intriguing aspect is the specificity of ChREBP upregulation according to tumor etiology. Indeed, different oncogenic pathways may predominate depending on the causative agent [14], and the identification of those leading to ChREBP transcriptional upregulation is certainly a matter of interest. Furthermore, the histological evaluation of ChREBP activation status, that is, its nucleo/cytoplasmic distribution in tumoral and surrounding nontumoral tissues, may be of prognostic value and even inform on the potential sensitivity or resistance to multikinase inhibitor‐based therapies. Metabolic reprogramming in tumor cells helps them to escape from immune surveillance by affecting the activation and cytotoxic functions of tumor‐associated immune cells [15]. Whether ChREBP‐mediated metabolic rewiring might favor the establishment of immune‐suppressive conditions is indeed worth exploring. In this context, the million‐dollar question would be whether ChREBP pharmacological inhibition could also increase the therapeutic response to ICI combination therapies. In any case, the work of Benichou and colleagues is a significant step forward in the path from sweet dreams to reality.

## Conflict of interest

The authors declare no conflict of interest.

## Author contributions

MGFB and MAA made equal contributions to this commentary.
